# Food Reputation and Food Preferences: Application of the Food Reputation Map (FRM) in Italy, USA, and China

**DOI:** 10.3389/fpsyg.2020.01499

**Published:** 2020-07-14

**Authors:** Stefano De Dominicis, Flavia Bonaiuto, Ferdinando Fornara, Uberta Ganucci Cancellieri, Irene Petruccelli, William D. Crano, Jianhong Ma, Marino Bonaiuto

**Affiliations:** ^1^Department of Nutrition, Exercise and Sports, University of Copenhagen, Copenhagen, Denmark; ^2^Dipartimento di Medicina Sperimentale, Sapienza Università di Roma, Rome, Italy; ^3^Facoltà di Economia, Universitas Mercatorum, Rome, Italy; ^4^Dipartimento di Pedagogia, Psicologia, Filosofia, Università degli Studi di Cagliari, Cagliari, Italy; ^5^CIRPA—Centro Interuniversitario di Ricerca in Psicologia Ambientale, Rome, Italy; ^6^Università per Stranieri “Dante Alighieri” di Reggio Calabria, Reggio Calabria, Italy; ^7^Department of Psychology, Claremont Graduate University, Claremont, CA, United States; ^8^School of Psychology and Behavioural Sciences, Zhejiang University, Hangzhou, China; ^9^Dipartimento di Psicologia dei Processi di Sviluppo e Socializzazione, Sapienza Università di Roma, Rome, Italy

**Keywords:** food reputation map, reputation, food preferences, consumer behavior, cultural differences, food choices, measure, food behavior

## Abstract

Given the food challenges that society is facing, we draw upon recent developments in the study of how food reputation affects food preferences and food choices, providing here a starting standard point for measuring every aspect of food reputation in different cultural contexts across the world. Specifically, while previous attempts focused either on specific aspects of food or on measures of food features validated in one language only, the present research validates the Food Reputation Map (FRM) in Italian, English and Chinese over 2,250 participants worldwide. Here we successfully measure food reputation across 23 specific indicators, further grouped into six synthetic indicators of food reputation. Critically, results show that: (a) the specific measurement tool of food reputation can vary across cultural contexts, and that (b) people's reputation of food products or categories changes significantly across different cultural contexts. Therefore, in order to understand people's food preferences and consumption, it is important to take into account the repertoire of cultural differences that underlies the contexts of analysis: the three context-specific versions of the FRM presented here effectively deal with this issue and provide reliable context-specific insights on stakeholders' interests, perspectives, attitudes and behaviors related to food perceptions, assessment, and consumption, which can be effectively leveraged to foster food sustainability.

## Introduction

The worldwide crisis regarding food and obesity poses a series of challenges to individuals, scholars, practitioners, policy makers, and society in general (Hawkes et al., 2015). For example, to face obesity, which is predicted to affect 51% of the population by 2030, interventions are required that can generate improvements at a systemic level (Finkelstein et al., 2012). Among other possibilities, a social-psychological approach aimed at understanding why and how consumers make certain food choices could be a promising tactic to initiate such interventions. Based on collectively shared judgements about a given entity (Moscovici, 1988), the concept of reputation may shed light on a series of social-psychological processes that individuals use in their transactions with food. Because reputation is a concept that affects and orients knowledge, trust, attitude, and choices toward a specific object, it can be considered both a social process and the product of such a process (Bonaiuto et al., 2017). Reputation is defined as the “the distribution of opinions about a person or other entity, in a stakeholder or interest group” (Bromley, 2001, p. 154), and, critically, it can be used to refer to any entity, such as individuals (Emler, 1990), groups (Bromley, 2001), organizations (Riel and van Fombrun, 2007), and even food (Bonaiuto et al., 2012b, 2017).

Past research has shown that perceived characteristics of food (related to food reputation), rather than objective ones (related to information), might exert a stronger effect on consumer choices (e.g., Carfora et al., 2019). Therefore, studying food reputation can potentially provide promising insights to implement systemic interventions that would help tackling the aforementioned food and obesity crisis, among other issues. Furthermore, the conceptualization of food as a social agent implies that food should be considered as a place-specific agent: food is basic, and as such it regards biology (physiological experience), psychology (individual experience), and culture (social experience) (Rozin, 2007).

According to this logic, the general contribution of the present work is 2-fold. First, drawing upon previous results (Bonaiuto et al., 2012b, 2017), it further refines the existing scale for measuring food reputation (the Food Reputation Map; Bonaiuto et al., 2017), helping the work in the understanding of food reputation. Second, in the current research FRM is validated in three languages—Italian (its original version), English, and Chinese, across their three respective cultural settings—contributing to the understanding of food as a social agent within cross-cultural lenses (Rozin, 2007). The present work could potentially inform future research on both individual and socio-cultural factors that drive food consumption and consequently stimulate discussion, applications and interventions toward a more sustainable and ethical food consumption (Vermeir and Verbeke, 2006).

### Defining Food Reputation

The rationale behind food reputation lies in the conceptualization of food as a social agent, since its features integrate both individual and collective processes (Conner and Armitage, 2002). Such a wide and open perspective, appropriate to understand food reputation, defines food features along three main areas (Bonaiuto et al., 2012b): (1) features linked to food (physical-chemical features and nutritional content); (2) features linked to the environment (economical, social and cultural features); and (3) features linked to the effects on the individual (sensorial, physiological and psychological effects). As such, food reputation can be considered both the social process of creating shared meaning around the concept of food and the result of such a process (Bonaiuto et al., 2017). Therefore, food reputation is defined as the whole set of beliefs (representations, attitudes, direct and indirect knowledge, etc.) that individuals hold about food. It includes beliefs about its antecedents and consequences (i.e., its production and its effects), its present features, its overall attractiveness (based on past direct and indirect experiences), and the future expectations related to its usage and consumption (Bonaiuto et al., 2012b, 2017). Based on this definition, an integrative model has been developed to operationalize food reputation's various facets. Established via a series of studies (qualitative, quantitative, and experimental) which indeed employed an international pool of experts (e.g., focus group) and participants (e.g., RCT experiment; see Bonaiuto et al., 2012a,b, 2017), the Food Reputation Map (henceforth, FRM) integrates the intrinsic characteristics of food, its effects on the environment, and its effects on the individual encompassing six main areas (second-order factors), namely “Synthetic Indicators” of food reputation: Essence, Cultural Effects, Economical Effects, Environmental Effects, Physical Effects, and Psychological Effects. These areas are further articulated into 23 specific areas (first-order factors), namely “Specific Indicators” of food reputation: Composition, Genuineness, Life time, Recognition, Territorial identity, Tradition, Familiarity, Innovativeness, Context, Price, Preparation, Social and environmental responsibility, Traceability, Proximity, Safety, Ability to satisfy, Digestibility, Lightness, Organoleptic perception, Personal memories, Psycho-physical well-being, Conviviality, Group belongingness.

On the basis of a series of different studies encompassing different methodological approaches—qualitative and quantitative, correlational and experimental (Bonaiuto et al., 2012a,b, 2017), these areas represent all the known possible features of food reputation.

### Food Reputation in Different Cultures

It is no secret that food and its processes vary dramatically across cultures (Rozin, 2007). The simple fact that cultural anthropology is the central discipline in the field of food and culture (Counihan and Van Esterik, 2013) exemplifies the relevance and complexity of this topic. Furthermore, considering that in the past 20 years, an enormous amount of research, scientific publications, books, websites, policies, and applied interventions have been dedicated to the social and cultural aspects of food and its consumption (Counihan and Van Esterik, 2013), it appears to be a matter of fact that any consideration related to the concept and processes of reputation cannot overlook the cultural differences that would affect it. Similarly, because of food's social agent nature (Bonaiuto et al., 2017), research on food, the individuals choosing it, food choice environments and food related processes cannot overlook the changing processes that food continuously undergoes (Devine, 2005).

Based on this reasoning, a fundamental question therefore inquires whether food reputation, as defined by the FRM model, would be different across different cultural settings. To test this, and to further validate the FRM model internationally, a series of three studies in three different cultural settings have been conducted.

### The Present Research

Drawing upon the FRM model (Bonaiuto et al., 2017), the present research validates the FRM questionnaire in three different cultural contexts. The resulting three validated versions of the FRM, namely FRM-ITA, FRM-ENG, and FRM-CHI (in Italian, English, and Chinese, respectively) are reported in Appendix 1. Three studies are presented. They were conducted between 2013 and 2016, in Italy first, and then in the U.S. and in China. In the three studies, the FRM has been tested with reference to the same three different goods—vegetables, peeled tomatoes and citrus fruit—chosen because they represented the three major food categories within the Italian economy (Castiglione et al., 2007; Zaccarini Bonelli, 2012) and to keep a comparable set of products.

#### Aims and Hypotheses

The aim of the three reported studies is to validate the measurement model for each language-specific version of the FRM initially presented by Bonaiuto et al. (2017), through a Confirmatory Factor Analysis performed via Structural Equation Modeling (SEM). Analyzing the factorial structure of the FRM using a CFA allows for testing both convergent and discriminant construct validity (Bagozzi et al., 1991; Corral-Verdugo, 2002). According to the presupposition that individuals' perceptions of food are culturally defined (Conner and Armitage, 2002; Rozin, 2007), we assume that the specific facets that operationalize food reputation (i.e., the most appropriate item-markers measuring the Specific Indicators) can differ across different cultures.

#### Operative Hypotheses

To verify such assumption, the operative hypotheses of Study 1, Study 2 and Study 3 concerned the adequacy of:

H1. The model fit indexes for each Synthetic Indicator of Food Reputation, which were modeled on the basis of both the initial theoretical constructs that generated the Specific Indicators of Food Reputation (Bonaiuto et al., 2012b, 2017) and the results of the principal component analyses in present and previous data (Bonaiuto et al., 2017);
H2. The correlations among Specific Indicators of Food Reputation (i.e., latent variables), in terms of size/statistical significance.
H3. The lambda coefficients connecting items (observed variables) and Specific Indicators of Food Reputation (latent variables), in terms of size/statistical significance.

#### Data Analysis

Reverse items were recoded such that higher scores always mean a positive reputation content. Then, data were analyzed through Confirmatory Factor Analysis via Structural Equation Modeling, to provide evidence for convergent and discriminant construct validity (Fornara et al., 2010). All analyses were conducted using the software STATA-14.

Across the three studies, the structural models performed to conduct the various CFAs validate the FRM-ITA, FRM-ENG, and FRM-CHI for each of the six Synthetic Indicators of food reputation. Following the approach of Hu and Bentler (1999) and Schreiber et al. (2006), three indexes to examine the model's goodness of fit are used here: the RMSEA, the SRMR, and the CFI, respectively with cut off values of 0.08, 0.08 (0.06 for close fit), and 0.95. Following previous research (Fornara et al., 2010), the RMSEA value was prioritized when deciding whether to accept the model. For each Synthetic Indicator of food reputation, a step-by-step iterative procedure was followed to modify the initial solution, including all items loading only on the expected factor (according to results of Bonaiuto et al., 2017). Both conceptual criteria (i.e., the retained sets of items reflected high content validity; see Fornara et al., 2010) and statistical criteria (i.e., statistical confirmation was provided by modification indexes analysis; Chou and Bentler, 1990) led to the emergent factorial solutions presented in the results section of each study. To ensure that the SEMs were identified, a constraint was added to the first indicator for each latent variable.

## Study 1: FRM-ITA

### Aims and Hypotheses

Study 1 applies the aims and hypotheses reported in section Aims and Hypotheses in the Italian context and therefore concerns the validation of the Italian version of the FRM, henceforth FRM-ITA.

### Methods

#### Participants and Procedure

The paper-and-pencil survey was administrated to a total of about 1,500 participants, from June to December 2013. Using a stratified sampling procedure, participants were recruited in public areas across Italy where they were individually asked to fill-in a 10–15-min survey about food for research purposes. Randomly, each participant was assigned to one of the three possible conditions (either vegetables, peeled tomatoes, or citrus fruit, as target food object). After a preliminary data screening (incomplete survey, response set, missing data), a finalized sample of *N* = 1,337 was used for data analysis. The finalized sample was evenly distributed across conditions (vegetables: 32.7%; peeled tomatoes: 34.3%; citrus fruit: 33%) and composed of: women 60.4%; Italians from North (26.4%), Center (31.4%), South (34.2%) and Major Islands (9.8%) of Italy; average age: 38.3 years (*SD*: 14.6); lower education (9.8%), high school (53.9%), university degree (31.2%), post-graduate education (2.2%). Importantly, participants' Body Mass Index (underweight: 4.5%; healthy weight: 66.7%; overweight: 24.4%; obese: 4.4%) was similar to a comparable sample of Italians (ISTAT, 2019), meaning that our sample is not biased about a food-related relevant index.

#### Measures

The FRM-ITA survey consists of 102 items measured on seven-point Likert-type scales (from “Completely disagree” to “Completely agree”); it was administrated in Italian. More specifically, the FRM-ITA tool includes 10 *General Food Reputation* items (Bonaiuto et al., 2017), the first of which focuses on the general reputation of the evaluated product, and the remaining on product (the target object) and process (how it is created) reputation. The FRM-ITA also includes 92 items devoted to measure the 23 Specific Indicators (originally containing 4-items each) of food reputation on separate scales. In the food reputation section, participants rated each item according to the following instruction (where, for each given questionnaire, X was substituted by the specific food item label to be assessed with FRM-ITA): “For each of the following statements, please indicate to what extent the reported characteristic describes X. It is enough to express your own opinion, on the basis of what you know about X, or according to whatever you have read, seen and heard about it.” This instruction aims to trigger the reputation framework judgment of a target entity based on an individual's both direct and indirect experience of it.

The final section of the self-report questionnaire included six items that assessed gender, age, education, area of origin in Italy, height, and weight.

### Results and Discussion

Following the FRM model, results of the first-order CFAs show the best measurement models (item-markers) for each Specific Indicator of food reputation: fit indices for each model measured by the FRM-ITA (H1), covariances among Specific Indicators for each Synthetic Indicator of food reputation (H2), and lambda coefficients for the retained items (H3) are reported in Tables 1, 4–9, **22**, respectively.

**Table 1:** CFA on food reputation's synthetic indicators of FRM-ITA–Study 1.

**Goodness of fit indexes**	**Chi^**2**^**	**df**	***p***	**RMSEA**	**SRMR**	**CFI**
Essence	354.71	46	<0.001	0.071	0.051	0.950
Cultural effects	274.86	56	<0.001	0.055	0.046	0.959
Economic effects	126.72	43	<0.001	0.080	0.070	0.960
Environmental effects	350.38	54	<0.001	0.065	0.064	0.949
Physiological effects	238.99	28	<0.001	0.076	0.043	0.953
Psychological effects	881.85	118	<0.001	0.071	0.068	0.943

*CFA, confirmatory factor analysis; RMSEA, root mean square error of approximation; SRMR, standardized root mean square residual; CFI, comparative fit index*.

**Table 2:** CFA on food reputation's synthetic indicators of FRM-ENG–Study 2.

**Goodness of fit indexes**	**Chi^**2**^**	**df**	***p***	**RMSEA**	**SRMR**	**CFI**
Essence	227.35	88	<0.001	0.072	0.065	0.957
Cultural effects	106.02	46	<0.001	0.066	0.055	0.962
Economic effects	126.72	43	<0.001	0.080	0.070	0.960
Environmental effects	99.59	41	<0.001	0.069	0.063	0.955
Physiological effects	47.43	20	<0.001	0.067	0.054	0.986
Psychological effects	337.71	128	<0.001	0.074	0.079	0.951

*CFA, confirmatory factor analysis; RMSEA, root mean square error of approximation; SRMR, standardized root mean square residual; CFI, comparative fit index*.

**Table 3:** CFA on food reputation's synthetic indicators of FRM-CHI–Study 3.

**Goodness of fit indexes**	**Chi^**2**^**	**df**	***p***	**RMSEA**	**SRMR**	**CFI**
Essence	187.63	68	<0.001	0.076	0.080	0.929
Cultural effects	175.23	66	<0.001	0.073	0.069	0.912
Economic effects	74.41	31	<0.001	0.068	0.053	0.942
Environmental effects	82.44	44	<0.001	0.053	0.038	0.950
Physiological effects	117.39	41	<0.001	0.078	0.069	0.949
Psychological effects	173.08	84	<0.001	0.059	0.064	0.964

*CFA, confirmatory factor analysis; RMSEA, root mean square error of approximation; SRMR, standardized root mean square residual; CFI, comparative fit index*.

**Table 4:** Covariance matrix of the specific indicators of food reputation for the synthetic indicator ESSENCE of FRM-ITA–Study 1.

**Essence**	**1**.	**2**.	**3**.	**4**.
1. Composition	–			
2. Genuineness	0.49^***^	–		
3. Life time		0.21^***^	–	
4. Recognition	−0.65^***^	−0.69^***^		–

*** *p < 0.001; empty cells represent non-constrained covariances*.

**Table 5:** Covariance matrix of the specific indicators of food reputation for the synthetic indicator CULTURAL EFFECTS of FRM-ITA–Study 1.

**Cultural effects**	**5**.	**6**.	**7**.	**8**.
5. Territorial Identity	–			
6. Tradition	0.56^***^	–		
7. Familiarity	0.53^***^	0.57^***^	–	
8. Innovativeness	0.38^***^	0.36^***^	0.50^***^	–

*** *p < 0.001; empty cells represent non-constrained covariances*.

**Table 6:** Covariance matrix of the specific indicators of food reputation for the synthetic indicator ECONOMIC EFFECTS of FRM-ITA–Study 1.

**Economic effects**	**9**.	**10**.	**11**.
9. Context	–		
10. Price		–	
11. Preparation	0.46^***^	0.04^†^	–

† p < 0.10;

*** *p < 0.001; empty cells represent non-constrained covariances*.

**Table 7:** Covariance matrix of the specific indicators of food reputation for the synthetic indicator ENVIRONMENTAL EFFECTS of FRM-ITA–Study 1.

**Environmental effects**	**12**.	**13**.	**14**.	**15**.
12. Social and Environm. Resp.	–			
13. Traceability	−0.15^***^	–		
14. Proximity		0.44^***^	–	
15. Safety	−0.5^***^	0.29^***^	0.08^**^	–

** p < 0.01;

*** *p < 0.001; empty cells represent non-constrained covariances*.

**Table 8:** Covariance matrix of the specific indicators of food reputation for the synthetic indicator PHYSIOLOGICAL EFFECTS of FRM-ITA–Study 1.

**Physiological effects**	**16**.	**17**.	**18**.
16. Ability to Satisfy	–		
17. Digestibility	0.72^***^	–	
18. Lightness	−0.61^***^	−0.65^***^	–

*** *p < 0.001; empty cells represent non-constrained covariances*.

**Table 9:** Covariance matrix of the specific indicators of food reputation for the synthetic indicator PSYCHOLOGICAL EFFECTS of FRM-ITA–Study 1.

**Psychological effects**	**19**.	**20**.	**21**.	**22**.	**23**.
19. Organoleptic perception	–				
20. Personal memories	0.18^***^	–			
21. Psyco-physical well-being	0.17^***^	0.45^***^	–		
22. Conviviality	−0.64^***^			–	
23. Group belongingness		0.54^***^	0.50^***^		–

*** *p < 0.001; empty cells represent non-constrained covariances*.

#### Essence

For the scale measuring Essence, the model includes four correlated Specific Indicators of Food Reputation (each measured by three items): Composition, Genuineness, Life Time, and Recognition. The Composition and Life Time factors, as well as Life Time and Recognition factors, were not significantly correlated.

#### Cultural Effects

For the scale measuring Cultural Effects, the model includes four correlated Specific Indicators of Food Reputation: Territorial Identity (three items), Tradition (three items), Familiarity (three items), and Innovativeness (four items).

#### Economic Effects

For the scale measuring Economic Effects, the model includes three correlated Specific Indicators of food reputation: Context (three items), Price (three items), and Preparation (four items). The factors Context and Price were not significantly correlated.

#### Environmental Effects

For the scale measuring Environmental Effects, the model includes four correlated Specific Indicators of food reputation: Social and Environmental Responsibility (three items), Traceability (three items), Proximity (four items), and Safety (three items). The Social and Environmental Responsibility and the Proximity factors were not significantly correlated.

#### Physiological Effects

For the scale measuring Physiological Effects, the model includes three correlated Specific Indicators of food reputation: Ability to Satisfy (three items), Digestibility (four items), and Lightness (three items).

#### Psychological Effects

For the scale measuring Psychological Effects, the model includes five correlated Specific Indicators of food reputation: Organoleptic Perception (four items), Personal Memories (three items), Psycho-physical Well-being (four items), Conviviality (three items), and Group Belongingness (four items). The following correlations among factors were not statistically significant: Personal Memories-Conviviality, Psycho-physical Well-being-Conviviality, and Conviviality-Group Belongingness.

### Conclusion

Study 1 results, based on an extensive correlational survey conducted across Italy, overall confirm the measurement model theorized by the original FRM (Bonaiuto et al., 2017): the tested models produced good fit indexes (H1); first order factors (i.e., the Specific Indicators of Food Reputation) were all correlated to each other (H2) within the following Synthetic Indicators of Food Reputation: Cultural Effects and Physiological Effects; and, a total of 76 items (from the initial total of 92 items) were retained (H3). In the finalized FRM-ITA, one item was removed from each of the following Specific Indicators of Food Reputation (the relevant overarching group's Synthetic Indicator of Food reputation is indicated in brackets): Composition, Genuineness, Life time, Recognition (Essence); Territorial identity, Tradition, Familiarity (Cultural Effects); Context, Price (Economic Effects); Social and Environmental responsibility, Traceability, Safety (Environmental Effects); Ability to satisfy, Lightness (Physiological Effects); Personal memories, Conviviality (Psychological Effects). The finalized FRM-ITA is given in Appendix 1. In conclusion, in Study 1 the FRM-ITA results successfully achieved. Hence, the English version of the instrument is targeted.

## Study 2: FRM-ENG

### Aims and Hypotheses

Study 2 applies the aims and hypotheses reported in section Aims and Hypotheses in the U.S. context, and therefore is concerned with the validation of the English version of the FRM, henceforth FRM-ENG. It originates from the FRM-ITA, which was translated and then back-translated by a team of English and Italian native speaker scholars (including some of the authors).

### Method

#### Participants and Procedure

The online survey was administrated to a total amount of about 400 participants, during October 2016. Participants were recruited in the USA using Amazon's Mechanical Turk (MTurk), a crowd sourcing website that allows the public to complete a variety of tasks, such as research studies. Studies using MTurk are valid and reliable (Rand, 2012; Siegel et al., 2019) and allow to reach a more demographically diverse sample (Buhrmester et al., 2011). In this study, participants volunteered to participate in a 10–15-min survey about food for research purposes. Similar to Study 1, each participant was randomly allocated to one of the three possible conditions (vegetables, peeled tomatoes, citrus fruit). After a preliminary data screening (incomplete survey, response set, missing data), a finalized sample (*N* = 303) was used for data analysis. The finalized sample was evenly distributed across conditions (vegetables: 33.3%; peeled tomatoes: 33.0%; citrus fruit: 33.7%) and composed of: women 52.1%; Americans: 93.4%; average age: 36.9 years (*SD*: 11.9); high school = 34.7%, bachelor degree = 48.2%, master degree = 12.9%, post-graduate education = 4.3%; employed full time = 62.7%, part time = 12.2%, unemployed = 8.9%, student = 5.3%, retired = 2.6%, occasional job = 2.3%, other = 5.6%; married = 47.2%, single = 35.6%, cohabitee or in a relationship = 8.6%; separated or divorced = 7.9%, widow/a = 1%. Similar to USA national data (DNPAO, 2019), where about half the people are overweight and obese, participants' Body Mass Index was: underweight, 3.8%; healthy weight, 46.4%; overweight, 29.4%; obese, 20.5%.

#### Measures

The FRM-ENG survey consists of the same 102 items used in the FRM-ITA (see section Method for details). The latter has been translated in English and back-translated in Italian. All items were measured on seven-point Likert-type items (from “Completely disagree” to “Completely agree”). The final section of the questionnaire included eight items measuring gender, age, education, marital status, employment, nationality, height and weight.

### Results and Discussion

Following the FRM model, results of the first-order CFAs show the best measurement models (item-markers) for each Specific Indicator of food reputation: fit indices for each model measured by the FRM-ENG (H1), covariances among Specific Indicators for each Synthetic Indicator of food reputation (H2), and lambda coefficients for the retained items (H3) are reported in Tables 2, 10–15, **22**, respectively.

**Table 10:** Covariance matrix of the specific indicators of food reputation for the synthetic indicator ESSENCE of FRM-ENG–Study 2.

**Essence**	**1**.	**2**.	**3**.	**4**.
1. Composition	–			
2. Genuineness	0.97^***^	–		
3. Life time	0.29^***^	0.34^***^	–	
4. Recognition	0.96^***^	0.96^***^	0.33^***^	–

*** *p < 0.001; empty cells represent non-constrained covariances*.

**Table 11:** Covariance matrix of the specific indicators of food reputation for the synthetic indicator Cultural effects of FRM-ENG–Study 2.

**Cultural Effects**	**5**.	**6**.	**7**.	**8**.
5. Territorial identity	–			
6. Tradition	−0.75^***^	–		
7. Familiarity	−0.49^***^	0.71^***^	–	
8. Innovativeness	0.24^***^	−0.49^***^	−0.77^***^	–

*** *p < 0.001; empty cells represent non-constrained covariances*.

**Table 12:** Covariance matrix of the specific indicators of food reputation for the synthetic indicator ECONOMIC EFFECTS of FRM-ENG–Study 2.

**Economic effects**	**9**.	**10**.	**11**.
9. Context	–		
10. Price	−0.41^***^	–	
11. Preparation	0.45^***^	−0.56^***^	–

*** *p < 0.001; empty cells represent non-constrained covariances*.

**Table 13:** Covariance matrix of the specific indicators of food reputation for the synthetic indicator ENVIRONMENTAL EFFECTS of FRM-ENG–Study 2.

**Environmental effects**	**12**.	**13**.	**14**.	**15**.
12. Social and Environm. Resp.	–			
13. Traceability	−0.60^***^	–		
14. Proximity	−0.24^***^		–	
15. Safety	−0.64^***^	−0.61^***^		–

*** *p < 0.001; empty cells represent non-constrained covariances*.

**Table 14:** Covariance matrix of the specific indicators of food reputation for the synthetic indicator PHYSIOLOGICAL EFFECTS of FRM-ENG–Study 2.

**Physiological effects**	**16**.	**17**.	**18**.
16. Ability to satisfy	–		
17. Digestibility	0.51^***^	–	
18. Lightness	−0.57^***^	−0.87^***^	–

*** *p < 0.001; empty cells represent non-constrained covariances*.

**Table 15:** Covariance matrix of the specific indicators of food reputation for the synthetic indicator PSYCHOLOGICAL EFFECTS of FRM-ENG–Study 2.

**Psychological effects**	**19**.	**20**.	**21**.	**22**.	**23**.
19. Organoleptic perception	–				
20. Personal memories		–			
21. Psyco-physical well-being	0.39^***^	0.37^***^	–		
22. Conviviality	−0.76^***^		−0.37^***^	–	
23. Group belongingness		0.60^***^	0.39^***^		–

*** *p < 0.001; empty cells represent non-constrained covariances*.

#### Essence

For the scale measuring Essence, the model includes four correlated Specific Indicators of food reputation (factors), each measured by four items: Composition, Genuineness, Life Time, and Recognition.

#### Cultural Effects

For the scale measuring Cultural Effects, the model includes four correlated Specific Indicators of food reputation, each measured by three items: Territorial Identity, Tradition, Familiarity, and Innovativeness.

#### Economic Effects

For the scale measuring Economic Effects, the model includes three correlated Specific Indicators of food reputation, each measured by four items: Context, Price, and Preparation.

#### Environmental Effects

For the scale measuring Environmental Effects, the model includes four correlated Specific Indicators of food reputation, each measured by three items: Social and Environmental Responsibility, Traceability, Proximity, and Safety. The factors Traceability and Proximity, and Proximity and Safety were not significantly correlated.

#### Physiological Effects

For the scale measuring Physiological Effects, the model includes three correlated Specific Indicators of food reputation, each measured by three items: Ability to Satisfy, Digestibility, and Lightness.

#### Psychological Effects

For the scale measuring Psychological Effects, the model includes five correlated Specific Indicators of food reputation: Organoleptic Perception (four items), Personal Memories (three items), Psycho-physical Well-being (three items), Conviviality (three items), and Group Belongingness (four items). The correlations among the following factors were not statistically significant: Organoleptic Perception-Personal Memories, Organoleptic Perception-Group Belongingness, Personal Memories-Conviviality, and Conviviality-Group Belongingness.

### Conclusion

Similar to Study 1, results of Study 2, based on a survey administered to an *ad hoc* sample of North Americans confirm the measurement model theorized by the original FRM (Bonaiuto et al., 2017): the hypothesized models produced good fit indexes H1); first order factors were all correlated to each other (H2) in the following Synthetic Indicators of Food Reputation: Essence, Cultural Effects, Economic Effects, and Physiological Effects; and, a total of 78 items were retained (H3). In the finalized FRM-ENG, one item was removed from each of the following Specific Indicators of Food Reputation (the group's Synthetic Indicator of Food reputation is indicated in brackets): Territorial identity, Tradition, Familiarity, Innovativeness (Cultural Effects); Social and environmental responsibility, Traceability, Proximity, Safety (Environmental Effects); Ability to satisfy, Digestibility, Lightness (Physiological Effects); Personal memories, Psycho-physical Well-being, Conviviality (Psychological Effects). The finalized FRM-ENG is presented in Appendix 1. In conclusion, Study 2 successfully defined the FRM-ENG. The Mandarin Chinese version of the instrument then was addressed.

## Study 3: FRM-CHI

### Aims and Hypotheses

Study 2 applies the aims and hypotheses reported in section Aims and Hypotheses in the U.S. context, and therefore is concerned with the validation of the Chinese-Mandarin version of the FRM, henceforth FRM-CHI. It originates from the FRM-ENG, which was translated and then back-translated by a team of English and Chinese-Mandarin native speakers scholars (including some of the authors).

### Method

#### Participants and Procedure

The online survey was administrated to about 350 participants, during May-August 2015. Participants were recruited at Zhejiang University (Hangzhou, China) via email using an available mailing list of students; to further populate the sample, participants were also recruited in the streets around the University and surveys were administered either via a mobile device or filled out in paper-and-pencil form. Respondents volunteered to participate in a 10–15-min survey about food for research purposes. Similar to Study 1 and 2, each participant was randomly allocated to one of the three possible conditions (vegetables, peeled tomatoes, citrus fruit). After a preliminary data screening (incomplete survey, response set, missing data), a finalized sample (*N* = 308) was used for data analysis. The finalized sample was evenly distributed across conditions (vegetables: 36.0%; peeled tomatoes: 31.5%; citrus fruit: 32.5%) and composed of: women 58.1%; Chinese: 100%; average age: 26.7 years (*SD*: 6.8); high school = 7.2%, bachelor degree = 79.4%, master degree = 10.8%, post-graduate education = 2.6%; married = 21.2%, single = 75.2%, cohabitee or in a relationship = 2.6%; separated or divorced = 0.3%, widow/er = 0.6%. Similar to Chinese national data (WHO, 2019), where only a small minority of inhabitants are obese, participants' Body Mass Index was: underweight, 21.2%; healthy weight, 69%; overweight, 6.1%; obese, 3.7%.

#### Measures

The FRM-CHI survey consists of the same 102 items used in the FRM-ENG (see section Method for details). The latter was translated in Chinese and then back-translated in English by a team of experienced researchers who were native in one language and fluent at the professional level in the other one (supervised by some of the co-authors). All items were measured on seven-point Likert-type items (from “Completely disagree” to “Completely agree”).

### Results and Discussion

As per Study 1–2, results of the first-order CFAs show the best measurement models (item-markers) for each Specific Indicator of food reputation: fit indices for each model measured by the FRM-CHI (H1), covariances among Specific Indicators for each Synthetic Indicator of food reputation (H2), and lambda coefficients for the retained items (H3) are reported in Tables 3, 16–21, 22, respectively.

**Table 16:** Covariance matrix of the specific indicators of food reputation for the synthetic indicator ESSENCE of FRM-CHI–Study 3.

**Essence**	**1**.	**2**.	**3**.	**4**.
1. Composition	–			
2. Genuineness	0.68^***^	–		
3. Life time	0.30^***^	0.11^***^	–	
4. Recognition	0.72^***^	0.57^***^		–

*** *p < 0.001; empty cells represent non-constrained covariances*.

**Table 17:** Covariance matrix of the specific indicators of food reputation for the synthetic indicator CULTURAL EFFECTS of FRM-CHI–Study 3.

**Cultural effects**	**5**.	**6**.	**7**.	**8**.
5. Territorial identity	–			
6. Tradition	0.22^***^	–		
7. Familiarity	0.53^***^		–	
8. Innovativeness	0.29^***^	0.20^***^	0.42^***^	–

*** *p < 0.001; empty cells represent non-constrained covariances*.

**Table 18:** Covariance matrix of the specific indicators of food reputation for the synthetic indicator ECONOMIC EFFECTS of FRM-CHI–Study 3.

**Economic effects**	**9**.	**10**.	**11**.
9. Context	–		
10. Price	0.39^***^	–	
11. Preparation	0.36^***^	0.42^***^	–

*** *p < 0.001; empty cells represent non-constrained covariances*.

**Table 19:** Covariance matrix of the specific indicators of food reputation for the synthetic indicator ENVIRONMENTAL EFFECTS of FRM-CHI–Study 3.

**Environmental effects**	**12**.	**13**.	**14**.	**15**.
12. Social and Environm. Resp.	–			
13. Traceability	−0.73^***^	–		
14. Proximity		0.30^***^	–	
15. Safety	−0.42^***^	0.44^***^	0.13^†^	–

† p < 0.10;

*** *p < 0.001; empty cells represent non-constrained covariances*.

**Table 20:** Covariance matrix of the specific indicators of food reputation for the synthetic indicator PHYSIOLOGICAL EFFECTS of FRM-CHI–Study 3.

**Physiological effects**	**16**.	**17**.	**18**.
16. Ability to satisfy	–		
17. Digestibility	0.46^***^	–	
18. Lightness	−0.25^***^	−0.96^***^	–

*** *p < 0.001; empty cells represent non-constrained covariances*.

**Table 21:** Covariance matrix of the specific indicators of food reputation for the synthetic indicator PSYCHOLOGICAL EFFECTS of FRM-CHI–Study 3.

**Psychological effects**	**19**.	**20**.	**21**.	**22**.	**23**.
19. Organoleptic perception	–				
20. Personal memories		–			
21. Psyco-physical well-being	0.46^***^	0.38^***^	–		
22. Conviviality	−0.71^***^		0.46^***^	–	
23. Group belongingness		0.46^***^	0.28^***^		–

*** *p < 0.001; empty cells represent non-constrained covariances*.

**Table 22:** Standardized lambda coefficients for each item and its specific indicator of food reputation in each version of the FRM.

**Reputational area**	**Synthetic indicator**	**#**	**Specific indicator**	**Item label**	**FRM-ITA**	**FRM-ENG**	**FRM-CHI**
Area 0—Food intrinsic features	Essence	1.	Composition	comp1	0.68	0.85	0.81
				comp2		0.90	0.86
				comp3	−0.86	−0.53	
				comp4	0.69	0.71	0.73
		2.	Genuineness	genuin1	0.48	0.87	0.87
				genuin2	0.49	0.89	0.94
				genuin3		−0.54	0.32
				genuin4	−0.73	−0.68	−0.31
		3.	Life time	lifetime1	0.38	0.61	0.82
				lifetime2	−0.79	−0.78	−0.34
				lifetime3		0.63	0.24
				lifetime4	−0.74	−0.93	−0.21
		4.	Recognition	recog1		0.79	0.55
				recog2	0.63	−0.51	
				recog3	0.73	−0.56	−0.22
				recog4	0.81	−0.63	−0.42
Area 1—Food-context effects or relations	Cultural effects	5.	Territorial identity	terr_id1	0.40	0.29	0.17
				terr_id2	0.85	0.75	0.45
				terr_id3	0.73	0.93	0.84
				terr_id4			
		6.	Tradition	trad1		0.12	0.82
				trad2	0.77	−0.84	
				trad3	−0.21		0.76
				trad4	0.76	−0.74	−0.16
		7.	Familiarity	famil1		0.58	−0.40
				famil2	0.70		0.61
				famil3	−0.41	0.46	−0.48
				famil4	0.73	−0.73	0.77
		8.	Innovativeness	innov1	0.80	0.84	0.43
				innov2	0.76		0.76
				innov3	0.77	0.91	0.57
				innov4	0.81	0.86	0.80
	Economic effects	9.	Context	contex1	0.19	0.80	0.75
				contex2	−0.75	−0.39	−0.44
				contex3	−0.88	−0.47	
				contex4		0.82	0.54
		10.	Price	price1		0.63	
				price2	0.63	−0.44	0.76
				price3	0.72	−0.89	0.83
				price4	0.81	−0.92	0.70
		11.	Preparation	prep1	0.37	0.94	0.97
				prep2	−0.72	−0.45	−0.23
				prep3	0.43	0.78	0.61
				prep4	−0.81	−0.52	−0.25
	Environmental Effects	12.	Social and environmental responsibility	resp1	0.86	0.72	0.30
				resp2	0.83	0.99	
				resp3	−0.18	−0.29	−0.70
				resp4			−0.69
		13.	Traceability	traceab1		−0.93	
				traceab2	0.53	0.42	0.59
				traceab3	0.85	0.38	1.30
				traceab4	0.76		0.32
		14.	Proximity	prox1	0.93	0.47	0.68
				prox2	0.67	0.50	0.60
				prox3	−0.28		
				prox4	−0.29	0.29	0.28
		15.	Safety	saf1	0.27		0.90
				saf2		−0.40	0.83
				saf3	−0.85	0.93	−0.09
				saf4	−0.89	0.89	
Area 2—Food-individual effects or relations	Physiological effects	16.	Ability to satisfy	ab_satisfy1	0.27	0.63	0.88
				ab_satisfy2	−0.84	−0.95	−0.59
				ab_satisfy3	−0.28	0.78	−0.75
				ab_satisfy4			0.63
		17.	Digestibiliy	digest1	0.30	0.46	0.55
				digest2	0.22		0.40
				digest3	−0.82	−0.85	−0.42
				digest4	−0.81	−0.97	−0.53
		18.	Lightness	light1	0.78	0.87	0.33
				light2	0.80	0.88	0.51
				light3	−0.65	−0.56	−0.87
				light4			−0.80
	Psychological effects	19.	Organoleptic perception	perc1	0.52	0.71	0.58
				perc2	0.42	0.64	
				perc3	−0.78	−0.97	−0.81
				perc4	−0.79	−0.95	−0.92
		20.	Personal memories	memor1	0.80	0.75	0.66
				memor2			
				memor3	0.81	0.88	0.91
				memor4	0.88	0.87	0.86
		21.	Psycho-physical well-being	well-being1	0.69		0.82
				well-being2	0.82	0.86	0.92
				well-being3	0.84	0.88	0.80
				well-being4	0.80	0.80	
		22.	Conviviality	conviv1			
				conviv2	0.71	0.80	0.83
				conviv3	0.80	0.81	0.84
				conviv4	0.73	0.87	0.71
		23.	Group belongingness	group_bel1	0.69	0.88	0.80
				group_bel2	0.90	0.91	0.91
				group_bel3	0.86	0.86	
				group_bel4	0.86	0.87	0.68

#### Essence

For the scale measuring Essence, the model includes four correlated Specific Indicators of food reputation (factors): Composition (three items), Genuineness (four items), Life Time (four items), and Recognition (three items). The factors Life Time and Recognition were not significantly correlated.

#### Cultural Effects

For the scale measuring Cultural Effects, the model includes four correlated Specific Indicators of food reputation: Territorial Identity (three items), Tradition (three items), Familiarity (four items), and Innovativeness (four items).

#### Economic Effects

For the scale measuring Economic Effects, the model includes three correlated Specific Indicators of food reputation: Context (three items), Price (three items), and Preparation (four items).

#### Environmental Effects

For the scale measuring Environmental Effects, the model includes four correlated Specific Indicators of food reputation, each measured by three items: Social and Environmental Responsibility, Traceability, Proximity, and Safety. The factors Social and Environmental Responsibility and Proximity were not significantly correlated.

#### Physiological Effects

For the scale measuring Physiological Effects, the model includes three correlated Specific Indicators of food reputation, each measured by four items: Ability to Satisfy, Digestibility, and Lightness.

#### Psychological Effects

For the scale measuring Psychological Effects, the model includes five correlated Specific Indicators of food reputation, each measured by three items: Organoleptic Perception, Personal Memories, Psycho-physical Well-being, Conviviality, and Group Belongingness. The following correlations among factors were not significant: Organoleptic Perception-Personal Memories, Organoleptic Perception-Group Belongingness, Personal Memories-Conviviality, and Conviviality-Group Belongingness.

### Conclusion

Similar to Studies 1 and 2, results of Study 3, based on a survey administered to an *ad hoc* sample of Chinese, generally confirms the measurement model theorized by the original FRM (Bonaiuto et al., 2017): the proposed models produced good fit indices (H1); first order factors (i.e., the Specific Indicators of Food Reputation) were all correlated in the following Synthetic Indicators of Food Reputation: Cultural Effects, Economic Effects, and Physiological Effects; and, a total of 77 items were retained (H3). In the finalized FRM-CHI, one item was removed from each of the following Specific Indicators of Food Reputation (the overarching group's Synthetic Indicator of Food reputation is indicated in brackets): Composition, Recognition (Essence); Territorial identity, Tradition (Cultural Effects); Context, Price (Economic Effects); Social and environmental responsibility, Traceability, Proximity, Safety (Environmental Effects); Organoleptic Perception, Personal memories, Psycho-Physical Well-being, Conviviality, Group Belongingness (Psychological Effects). The finalized FRM-CHI is presented in Appendix 1. In conclusion, Study 3 successfully defined the FRM-CHI.

### Auxiliary Analysis

**Figure 1:**
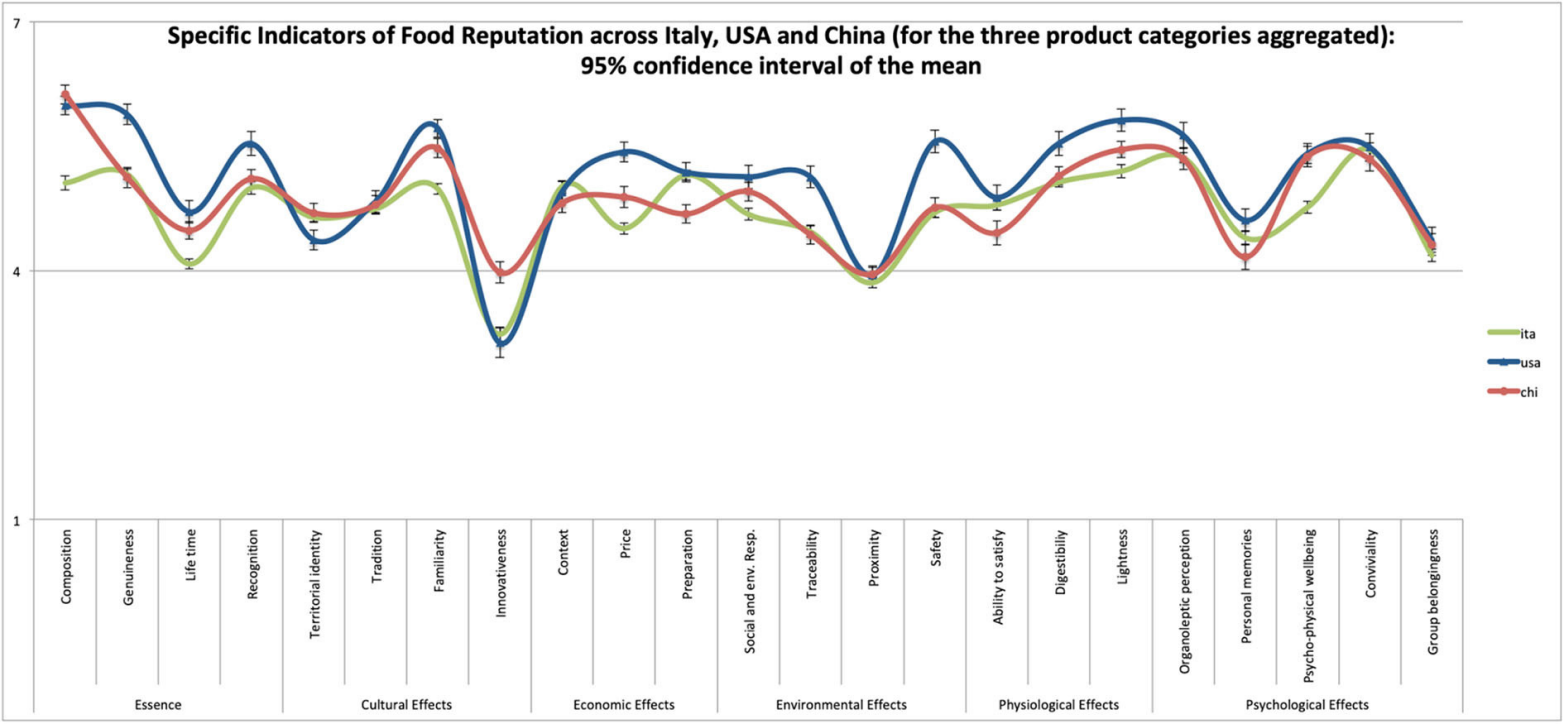
Statistical comparison of the three product categories aggregated across the three countries of Study 1, 2, and 3.

**Figure 2:**
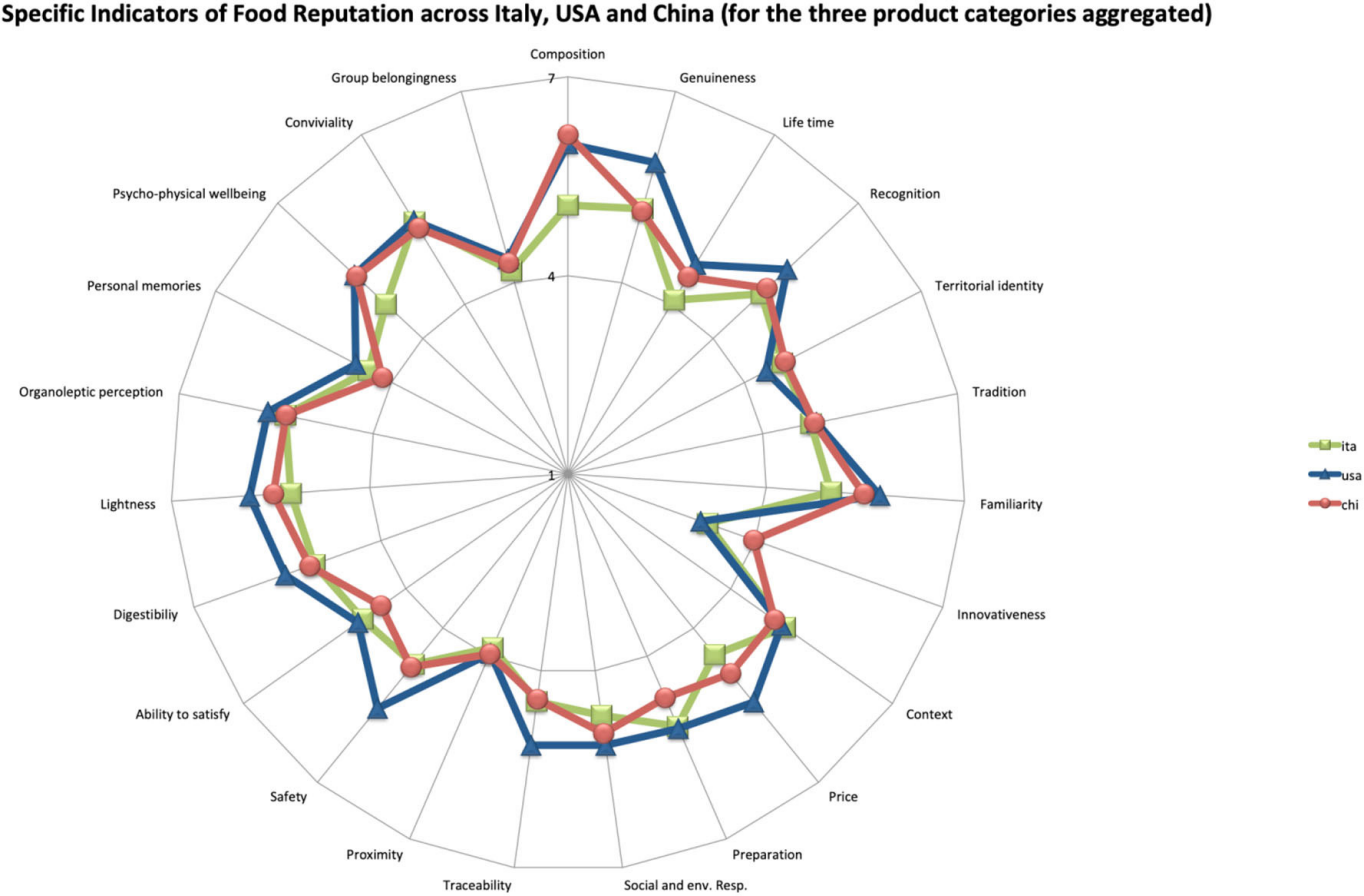
Kiviat graph for the descriptive representation of FRM.

To further corroborate the importance of defining the three versions of the FRM, we conducted a *post-hoc* auxiliary analysis where we compared the Specific Indicators of food reputation describing the three aggregated product categories in the three countries. A series of 23 ANOVAs has been conducted to test for significant differences in each Specific Indicator of food reputation (of the three food categories aggregated) across the three cultural contexts. Specifically, in Figure 1, we show the statistical differences (one-way ANOVA and 95% confidence interval of the mean) among the three countries for each specific indicator of food reputation. Results show that some reputational features of the examined food products are indeed perceived differently across cultures (all *p* < 0.05): for example, Italians perceive Composition, Life Time, Familiarity, Social and Environmental Responsibility, Lightness and Psycho-Physical Well-being to be significantly lower than Americans and Chinese; Americans perceive Genuineness, Recognition, Familiarity, Price, Social and Environmental Responsibility, Traceability, Safety, Digestibility and Lightness to be significantly higher than Italians and Chinese; Chinese perceive Preparation and Ability to Satisfy to be significantly lower, while Innovativeness significantly higher than Italians and Americans. Furthermore, as an example, we suggest the visual representation of the FRM in the form of a Kiviat graph (Morris, 1974)—or radar chart (Figure 2)—to have a visual representation of the magnitude of each given product category's reputational profile.

In conclusion, although the test of such statistical differences goes beyond the aim of the present research, we believe that such representation is a useful example to highlight one of the possible applications of the FRM. Results show that people's reputation of the food categories here examined changes significantly across different cultural contexts. These results, together with the fact that consumers increasingly demand local and traditional food (Pieniak et al., 2009), substantiate the idea that in order to understand people's actions in relation to food preferences and therefore food consumption, it is important to take into account the repertoire of cultural differences that underlies the contexts of analysis. In order to do so, the culture-specific measures of food-reputation here provided can therefore be very effective and useful tools to acquire such knowledge. In turn, the knowledge of culture-specific food perception could be indeed leveraged to promote a more sustainable consumption of food.

## General Discussion

The three reported studies succeeded in validating the original model proposed for the FRM (Bonaiuto et al., 2017). Drawing on the idea that food can play different roles in different cultures, and therefore the concept of food reputation might change across different cultural settings, it is important to provide available tools capable of measuring food related constructs cross-culturally. The three versions of the Food Reputation Map, namely the FRM-ITA, FRM-ENG, and FRM-CHI in Italian, American English, and Mandarin Chinese, respectively, were created and validated by administration of the same FRM item sets in Italy, USA and China (Table 1). Together, the three studies represent a first attempt at creating a series of tools that could be applied in future studies for improving the understanding of individuals' food perceptions, assessments, and consumption. Overall, the three studies confirm the measurement structure of the FRM through the verification of the three operational hypotheses of the present research: in fact, model fit indexes, correlations among Specific Indicators of food reputation, and lambda coefficient—only with few exceptions in each context—were satisfactory and confirmed the original theoretical model of food reputation already presented in past research (Bonaiuto et al., 2012b,c, 2017). The Food Reputation Map theoretical model, encompassing twenty-three Specific Indicators, which can be further grouped into six Synthetic Indicators of food reputation, was replicated in the three different cultural contexts, keeping constant three target food categories. The results held across three diverse samples of Italian, American, and Chinese respondents.

According to the assumptions that (a) food is a social agent (Bonaiuto et al., 2017), (b) it is fundamentally linked to specific cultural settings (Counihan and Van Esterik, 2013), and (c) is subject to continuous change (Devine, 2005), the three measures of food reputation presented here can be applied to study food reputation according to, and within, different cultural settings. The finalized sets of items measure each of the twenty-three Specific Indicators of food reputation either by a three-item or four-item marker pool: such items, in each cultural setting, apparently have a different weight in measuring the specific indicator of food reputation. Also, results show that correlations among Specific Indicators of food reputation—which, according to the FRM model cluster into a Synthetic Indicator of food reputation—can change across cultural settings. These results support the idea that food reputation can be measured by a standard set of items and can be synthesized by the same set of Indicators (Specific and Synthetic ones). This tenet does not exclude the possibility that specific target food categories can also be defined and rated differently according to different cultural settings in terms of their respective food reputation: further research should investigate which parameters of food reputation are, on the one hand, context specific and which are, on the other hand, generalizable across cultures.

### Limitations and Future Directions

The new and promising tools developed in this research should be considered in light of some limitations, which can guide future research developments for understanding both generalizable and context-specific features of food reputation. First, it should be noted that the new FRM measures developed here emerged from a testing involving specific samples in Italy, USA, and China (*N* = 1,337, 303, and 307, respectively), where participants responded to the specific survey in their own native language. Although the Italian sample was gathered by quota sampling in the Italian population across genders, ages, and main geographical areas, the American and Chinese samples were convenient samples (MTurk and college students, respectively). Whereas, the Italian sample could be considered representative of the Italian population (ISTAT, 2019) the American and Chinese sample might not be representative of their respective populations. Thus, future research should aim at replicating the FRM model within larger, representative sample within each context, possibly including various other socio-demographic information, such as ethnicity and immigration status to allow for a deeper understanding of how reputational features of a given food could be perceived differently within specific groups.

Second, the FRM tools developed in this research have referred to three different goods, namely vegetables, citrus fruit and peeled tomatoes, all very relevant goods for the Italian food market (Castiglione et al., 2007; Zaccarini Bonelli, 2012; Bonaiuto et al., 2017). However, these goods might not be central within other food markets in other cultural contexts, and therefore future research should consider how food reputation features are linked to products whose importance is more or less central within various specific cultural settings.

Also, as already pointed out in previous research (Bonaiuto et al., 2012b, 2017), the FRM model originated and has been developed from a set of initial researches (carried out with both qualitative and quantitative methodology) based in Italy (Bonaiuto et al., 2012a,b,c, 2017). Such a feature on the one hand is a strength considering the variety of approaches and methods used in its development; on the other hand, it could potentially represent a limiting factor in terms of cultural diversity. In fact, it is well-acknowledged that food is a fundamental aspect in the Italian culture (Parasecoli, 2004), and therefore it might be the case that some outcomes which emerged in the Italian sample could be culture-specific rather than cultural universals. Thus, a test of the FRM in other cultures is needed to generalize the validity of the FRM across different cultural and linguistic contexts, possibly considering classes of products, which are very relevant for those specific cultures, to assure the best benchmarking approach. Overall, the present research is a first attempt to set a standard measure, which can be used to assess this issue; however, future research should investigate whether other fundamental tenets of food reputation can arise in different contexts.

Furthermore, concerning factorial the structure of the FRM model, the second-order factors of food reputation (namely, the Synthetic Indicators) have not been discussed here—a goal that would have been out of the scope of the present manuscript. Rather, here we test which items of each Specific Indicator of food reputation (first-order factor) are indeed the most appropriate markers to measure the intended Specific Indicator within each culture. Future research should therefore confirm the second-order overall structure of the model in different cultural contexts. In addition, concerning the comparison between cultures, we assumed and demonstrated that the best item-markers for each Specific Indicator of food reputation can vary cross-culturally. However, one un-answered question is whether or not (and, if yes, which) facets of food reputation could be universally relevant: starting from the present results, future research should therefore test the multi-group invariance of the FRM model across different cultural groups.

### Practical Implications

In spite of such limitations, the present tools set an effective and useful standard of measures, which can be implemented in various practical activities. A series of possible applications can be considered in light of these new tools, which are highlighted here in view of future developments.

At the consumer level, one possible application lies in the opportunity to gather new knowledge on different reputational features, or perceived features that can be linked to reputation (Péneau et al., 2006), which may affect consumer choices in different contexts (Bonaiuto et al., 2012b). This goal could be achieved, for example, by investigating a given product's reputation in two different cultural contexts to understand its culture-specific reputational features' strengths and weaknesses. Such a strategy could be used to address various issues. For example, it could help individuals' decision-making on how to self-regulate eating behaviors (Johnson et al., 2012); or it could shed light on how ethnic identity, socialization and other culture-specific behaviors affect food consumption (Xu et al., 2004); or, in more general terms, it could deepen our understanding of cultural specific influences of food reputation on the attitude-behavior consistency (Crano and Prislin, 2011) related to food choices.

From a marketing perspective, because in both physical and online markets peer-to-peer knowledge represents a fundamental asset for consumers and businesses to derive information related to reputation and trust (Ert et al., 2016), the knowledge and management of a given food product's reputational features can obviously be an important asset. Drawing upon the evidence that different food reputational features have different impacts on consumers' food choices (Bonaiuto et al., 2012b,c), food reputation management could be a very effective strategy for various stakeholders (e.g., businesses or consumers) to gain strategic advantage from their competitors. On the one hand, businesses could, for example, grow their own reputation (Riel and van Fombrun, 2007) by taking advantage of their products' best reputational features, or they could improve their own products' reputation by investing in specific weak reputational features to be addressed. On the other hand, specific clusters of consumers, such as athletes or clinical patients, could acquire knowledge about specific reputational features of a given product and then use this knowledge to their advantage (for example, for improving performance or for sticking to prescribed nutritional programs; e.g., Johnson et al., 2012).

At the broader perspective, policy makers, opinion-leaders and various institutional stakeholders can potentially use the present tools to promote well-being at the community level. In fact, the various reputational features of the food reputation model, confirmed here, could be studied to serve purposes related to, among others, community-based health interventions (Schulz et al., 2005; Ball et al., 2010; Brand et al., 2014), environmental sustainability (Tilman and Clark, 2014), and price control (UN World Food Programme, 2012). Indeed, one of the major strengths of our research lies in the fact that by considering all possible facets of food reputation (defined by the FRM), and by developing a culture-specific instrument measuring such facets (e.g., FRM-ENG), it would be possible to understand whether a specific reputational feature (e.g., Tradition) could be leveraged to promote for example health or sustainable consumption at the community level in a given culture. We could draw upon the example of the Specific Indicator “Tradition”: although it has been often argued that there is no real *cuisine* tradition in the U.S. (e.g., Mintz, 2002), results of our Auxiliary analysis show no significant differences in Tradition across the three different cultures. This specific result could indicate that, despite that perhaps Italy and China have (at least historically) greater food traditions than the U.S., this is not reflected in the perceptions of individuals. Findings like this can inform marketers, practitioners, and policymakers alike to engage in more informed actions and solutions toward healthier, more sustainable and better informed food-related decisions.

Within this approach, the study of food reputational features could indeed be leveraged to promote food sustainability in a variety of ways. For example, provided that information about sustainability are communicated to consumers, consumption behaviors might have a major role in bringing about more sustainable food production (Grunert, 2011). Past research has already shown that some perceived features of food, such as food safety, environmental concern, nutritive value, taste, freshness and appearance—that are, arguably, very much comparable to the reputational features by the FRM, might influence organic food consumer preferences (Shafie and Rennie, 2012). However, how individuals can be encouraged to cut unsustainable consumption behavior (e.g., excessive meat consumption) has been underexplored, and more in-depth studies on the factors that could increase people's willingness to engage in a more sustainable food consumption are much needed (Hartmann and Siegrist, 2017). We argue that, ideally, the FRM model could be used to develop international evidence-based knowledge, which in turn could inform and support international exchange of information and effective policy design on drivers of sustainable consumer behavior and evaluations across countries worldwide (McGeevor, 2009).

### Conclusion

From a social-psychological perspective, understanding processes driving individuals' food preferences and food consumption (i.e., food consumer behavior), food markets, and political decision-making is an important asset to be developed. The fundamental importance of understanding the cultural specificity of food reputation (Parasecoli, 2004), is reflected in the assumption that human behavior can only exists in a given place, and therefore it is both the product of, and it produces, a whole series of transactions between individuals and the specific environments where their behavior occurs (Proshansky et al., 1970; Bronfenbrenner, 1977; Bonnes and Secchiaroli, 1995; Bonnes and Bonaiuto, 2002; Devine-Wright, 2013). The global issues and challenges related to food consumption must be faced by interdisciplinary efforts and tackled by multiple perspectives (FAO, 2009). On the one hand, a stunning 113 million people across 53 countries are suffering acute hunger (Global Report on Food Crises, 2019); on the other hand, trend forecasts suggest that by 2030, 51% of the population will be obese (Finkelstein et al., 2012). In this respect, the crucial importance of food reputation lies in both its theoretical and applied implications for understanding food consumption choices and behaviors. By drawing upon the present research program, and by further developing the measures provided, the detailed and specific knowledge capital that could be derived provides the initial building blocks for a number of new possible interventions and action plans designed to tackle the current global food–related challenges, and potentially be leveraged to foster food sustainability. From a behavioral science perspective, whether in the realm of the consumption, production, marketing, political, or clinical intervention over food and drink matters, a “think global, act local” approach (Devine-Wright, 2013) could materially facilitate the development of international sustainable solutions to some of the global challenges related to food.

## Data Availability Statement

The raw data supporting the conclusions of this article will be made available by the authors, without undue reservation.

## Ethics Statement

Ethical review and approval was not required for the study on human participants in accordance with the local legislation and institutional requirements. Written informed consent for participation was not required for this study in accordance with the national legislation and the institutional requirements.

## Author Contributions

SD and MB designed the research questions and the study. SD, MB, FF, WC, and JM supervised the data collection. SD performed the statistical analysis to discuss with MB and FF for interpretation and drafted the manuscript. WC, FF, and MB provided the feedback on the manuscript. SD, FB, UG, and IP contributed to the data collection. All authors contributed to the article and approved the submitted version.

## Conflict of Interest

The authors declare that the research was conducted in the absence of any commercial or financial relationships that could be construed as a potential conflict of interest.
