# Supplementary material for: Food Reputation and Food Preferences: Application of the Food Reputation Map (FRM) in Italy, USA, and China
Source: Front Psychol. 2020 Jul 14;11:1499. doi: 10.3389/fpsyg.2020.01499 (PMC7372101; doi:10.3389/fpsyg.2020.01499)
Supplement: Supplementary file 1 [file Data_Sheet_1.pdf]

Food reputation and food preferences: application of the Food Reputation Map (FRM) in Italy, USA and China

Stefano De Dominicis[1*], Flavia Bonaiuto[2-3], Ferdinando Fornara[4-5], Uberta Ganucci Cancellieri[6], Irene Petruccelli[3], William D. Crano[7], Jianhong Ma[8], Marino Bonaiuto[5-9]

[1] Department of Nutrition, Exercise and Sports, University of Copenhagen, Copenhagen, Denmark

[2] Dipartimento di Medicina Sperimentale, Sapienza Università di Roma, Roma, Italy.

[3] Facoltà di Economia, Universitas Mercatorum, Roma, Italy.

[4] Dipartimento di Pedagogia, Psicologia, Filosofia, Università degli Studi di Cagliari, Cagliari, Italy.

[5] CIRPA—Centro Interuniversitario di Ricerca in Psicologia Ambientale, Roma, Italy.

[6] Università per Stranieri "Dante Alighieri" di Reggio Calabria, Reggio Calabria, Italy.

[7] Department of Psychology, Claremont Graduate University, Claremont, CA, USA.

[8] School of Psychology and Behavioural Sciences, Zhejiang University, China.

[9] Dipartimento di Psicologia dei Processi di Sviluppo e Socializzazione, Sapienza Università di Roma, Roma, Italy.

*** Correspondence:**
Stefano De Dominicis
sdd@nexs.ku.dk

Keywords: Food Reputation Map, Reputation, Food preferences, Consumer behavior, Cultural differences, Food choices, Measure, Food behavior

Appendix 1

Appendix 1. FRM-ITA, FRM-ENG and FRM-CHI definition: Reputational Area, Synthetic Indicator of Food Reputation, Specific Indicator of Food Reputation, item label, items in Italian, American English and Chinese Mandarin. Items in bold are retained for the final version in each language. Items in italics are reverse coded items.

REPUTATIONAL AREA	SYNTHETIC INDICATOR	SPECIFIC INDICATOR	ITEM LABEL	ITALIAN	AMERICAN ENGLISH	CHINESE MANDARIN
Area 0 – Food Intrinsic Features	Essence	Composition	comp1	**Contengono proprietà nutritive importanti**	**They contain important nutritious properties**	它们含有重要的营养物质
			comp2	Sono nutrienti	**They are nutritious**	**它们是有营养的**
			comp3	***Apportano componenti di scarsa qualità***	***They provide poor nutrient quality***	*它们提供了较少的营养*
			comp4	**Hanno una composizione sana**	**They have an healthy composition**	它们有着健康的组成部分
		Genuineness	genuin1	**Sono genuini**	**They are natural**	它们是自然的
			genuin2	**Sono sani**	**They are healthy**	它们是健康的
			genuin3	*Contengono additivi chimici*	***They contain chemical additives***	*它们包含了化学添加剂*
			genuin4	***Contengono ingredienti artificiali***	***They contain artificial ingredients***	*它们包含了人工成分*
		Life time	lifetime1	**Deperiscono rapidamente**	**They quickly rot**	它们腐烂地很快

			lifetime2	***Si conservano bene nel tempo***	***They remain well preserved with time***	*它们可以保存得很久*
			lifetime3	Sono facilmente deteriorabili	**They easily decompose**	它们很容易分解
			lifetime4	***Non vanno a male velocemente***	***They do not rapidly deteriorate***	*它们不会很快地变质*
		Recognition	recog1	Sono riconoscibili	**They are recognizable**	它们很好辨识
			recog2	***Non si capisce cosa c'è dentro***	***It's hard to understand what they contain***	*很难以分辨出它们包含了什么*
			recog3	***Sono camuffati da altri sapori***	***They are masked by other flavours***	*它们被其他的味道掩盖了*
			recog4	***Sono di scarso valore***	***They are products of low value***	*它们是不太重要的食品*
Area 1 – Food-Context Effects or Relations	Cultural Effects	Territorial identity	terr_id1	**Sono legati a un territorio**	**They are bound to a territory**	它们是某一地区的特产
			terr_id2	***Non appartengono alla tradizione di un paese***	***They are detached from a country's traditions***	*它们和这个国家的传统相分离*
			terr_id3	***Non fanno parte di storia e costumi di una comunità***	***They are unrelated to the history and habits of a community***	*它们和这个群体的历史和习惯不相关*
			terr_id4	Hanno una loro identità territoriale	They have their own territorial identity	它们有它们自己的区域属性

		Tradition	trad1	Sono composti da ingredienti tradizionali	**They are made of traditional ingredients**	它们是由传统的方式种植的
			trad2	***Sono distanti dalla tradizione***	***They are far-removed from the tradition***	*它们和传统相分离*
			trad3	**Sono preparati secondo canoni tradizionali**	They are prepared according to traditional methods	它们是按照传统的方法制作的
			trad4	***Non rispecchiano usanze tradizionali***	***They are unrelated to traditions***	*它们和传统不相关*
		Familiarity	famil1	Sono familiari	**They are familiar**	它们是熟悉的
			famil2	***Non rientrano nelle abitudini personali***	*They are unrelated to personal habits*	*它们不符合我的饮食习惯*
			famil3	**Hanno un sapore familiare**	**They have a familiar taste**	它们有着熟悉的味道
			famil4	***Sono in contrasto con i gusti personali***	***They conflict with personal well known taste***	*它们和人们熟知的味道相矛盾*
		Innovativeness	innov1	**Hanno un sapore nuovo**	**They have a new taste**	它们有新的口味
			innov2	**Hanno un sapore sempre diverso**	They always taste novel	它们尝起来总是很新奇
			innov3	**Hanno un aspetto nuovo**	**They have a new appearance**	它们有新的外观

			innov4	**Hanno una consistenza diversa dal solito**	**Their consistence is different from usual**	它们总是与众不同
	Economic Effects	Context	contex1	**Vengono acquistati e/o consumati in un locale pulito**	**They are bought and/or consumed in a clean place**	它们是在一个干净的地方购买的/消费的
			contex2	***Vengono acquistati e/o consumati in un locale di dubbia fama***	***They are bought and/or consumed in a place of uncertain reputation***	*它们是在一个不知道怎样的地方购买的/消费的*
			contex3	***Vengono acquistati e/o consumati in un ambiente sgradevole***	***They are bought and/or consumed in an unpleasant place***	*它们是在一个不太愉快的地方购买的/消费的*
			contex4	Vengono acquistati e/o consumati in un locale con un'immagine positiva	**They are bought and/or consumed in a place with a positive image**	它们是在一个有着正面形象的地方购买的/消费的
		Price	*price1*	*Costano molto*	***They are expensive***	*它们是昂贵的*
			price2	**Valgono i soldi spesi per acquistarli**	**They worth the price**	它们值得这个价钱

			price3	**Hanno un prezzo adeguato alla loro effettiva qualità**	**They have an appropriate price for the quality**	它们有着符合质量的价格
			price4	**Hanno un buon rapporto qualità/prezzo**	**They have a good price/quality ratio**	它们性价比很高
		Preparation	prep1	**Sono semplici da preparare**	**They are easy to prepare**	它们很容易制作
			prep2	***Richiedono una preparazione elaborata***	***They require elaborate preparation***	*它们需要精心制作*
			prep3	**Sono veloci da preparare**	**They are quick to prepare**	它们可以快速被准备好
			prep4	***Richiedono tempi lunghi di preparazione***	***They take long time to be prepared***	*它们需要花很长的时间来准备*
	Environmental Effects	Social and environmental responsibility	*resp1*	***Producono elevati costi ambientali***	***They produce high environmental costs***	*它们造成了高昂的环境代价*
			resp2	***Hanno ripercussioni negative sull'ambiente***	***They have a negative impact on the environment***	*它们对环境有负面的影响*
			resp3	**Sono prodotti con metodi che rispettano l'ambiente**	**They are produced in a way that respects the environment**	它们是以一种尊重环境的方式制造的

			resp4	Sono prodotti in modo equo e solidale	They are produced in a fair trade way	它们是以一种公平的方式被交易
		Traceability	*traceab1*	*Non si sa da dove provengono*	***Their origin is unknown***	*它们的原产地不明*
			traceab2	**Hanno una provenienza tracciabile**	**They have a track able origin**	它们有着可追溯的产地
			traceab3	**Hanno un'origine garantita**	**They have an official origin**	它们有一个官方的产地
			traceab4	**Sono certificati**	They are certified	它们是有许可证的
		Proximity	prox1	**Sono della zona in cui si vive**	**They come from and are consumed in a specific place of residence**	它们在一个特定的地方产生和被消费
			prox2	**Sono "a chilometro zero"**	**They are farm-to-fork (local)**	它们是产地直供的
			prox3	***Non sono un prodotto locale***	*They are consumed in places that differ from where they originate*	*它们在与它们产地不一样的地方被消费*
			prox4	***Hanno origine in un luogo diverso da quello in cui li si consuma***	***They are from a place different from where they are consumed***	*它们不是产自被消费的地方*

		Safety	saf1	**Vengono controllati per stabilirne la sicurezza**	They are checked in order to establish their safety	它们经过了检验以保证安全
			saf2	Sono sottoposti a controlli di sicurezza alimentare	**They are subjected to food safety inspection**	它们受到了食品安全检验
			saf3	***Non sono sicuri***	***They are dangerous to consume***	*消费它们是危险的*
			saf4	***Non rispettano le normative sulla sicurezza alimentare***	***They violate food safety's regulations***	*它们违反了食品安全规定*
Area 2 - Food-Individual Effects or Relations	Physiological Effects	Ability to satisfy	ab_satisfy1	**Sono una fonte efficace di sostentamento**	**They satisfy hunger**	它们能充饥
			ab_satisfy2	***Sono poco utili all'organismo***	***They leave people hungry***	*它们不能消除人们的饥饿感*
			ab_satisfy3	***Non saziano pienamente***	***They do not fill people enough***	*它们不能填饱人们*
			ab_satisfy4	Appagano sul piano della sazietà	They satisfy in terms of satiation	它们能满足人们的饱腹感
		Digestibiliy	digest1	**Sono facilmente digeribili**	**They are easily digestible**	它们很容易被消化
			digest2	**Sono leggeri per lo stomaco**	They are light on the stomach	它们对胃的负担很小

			digest3	***Lasciano postumi fisici negativi***	***They leave negative physical consequences***	*它们有不好的生理影响*
			digest4	***Hanno ripercussioni negative sull'organismo***	***They have a negative impact on the body***	*它们对身体有不好的影响*
		Lightness	*light1*	***Fanno aumentare eccessivamente di peso***	***They make people gain excessive weight***	*它们让人们体重增加过量*
			light2	***Fanno ingrassare***	***They make people overweight***	*它们使人超重*
			light3	**Contribuiscono alla salute fisica**	**They contribute positively to physical health**	它们对身体健康有益
			light4	Contribuiscono a prevenire le malattie	They contribute to the prevention of diseases	它们有助于预防疾病
	Psychological Effects	Organoleptic perception	perc1	**Hanno un buon sapore**	**They taste good**	它们尝起来不错
			perc2	**Sono gustosi**	**They are appetizing**	它们能增加食欲
			perc3	***Hanno un sapore sgradevole***	***They have an unpleasant taste***	*它们有种让人不悦的味道*
			perc4	***Non soddisfano il palato***	***They do not taste good***	*它们尝起来不好*
		Personal memories	memor1	**Ricordano il passato**	**They remind people of the past**	它们让人想起过去

			memor2	*Non stimolano dei ricordi*	*They are not connected with memories of the past*	*它们与过去的记忆没有联系*
			memor3	**Fanno ricordare di quando si era bambini**	**They remind people of their childhood**	它们使人想起童年
			memor4	**Ricordano sapori e immagini di un tempo lontano**	**They remind people of flavors and images of the distant past**	它们让人想起很久以前的味道和印象
		Psycho-physical wellbeing	wellbeing1	**Promuovono il benessere psicofisico**	They promote mental and physical wellness	它们促进身心健康
			wellbeing2	**Incidono positivamente sull'umore**	**They positively affect mood**	它们对情绪有积极的作用
			wellbeing3	**Rigenerano dal punto di vista psicofisico**	**They revitalize mental and physical wellness**	它们使身心重现活力
			wellbeing4	**Provocano sentimenti ed emozioni positive**	**They elicit positive feelings and emotions**	它们激发了积极的感受和情绪
		Conviviality	conviv1	Possono essere consumati insieme ad altre persone	They can be consumed together with other people	它们可以和他人一起分享

			conviv2	***È difficile goderne in compagnia***	***It is not suitable to enjoy them in company***	*它们不适合一起享用*
			conviv3	***Rendono difficile la comunicazione con le altre persone***	***They make communication with other people hard***	*它们使与他人交流变得困难*
			conviv4	***Non si prestano a situazioni di socialità***	***They are not very suitable with social situations***	*它们在社交场合不是很适宜*
		Group belongingness	group_bel1	**Fanno sentire di appartenere alla propria famiglia**	**They make people feel like they belong to one's own family**	它们使人们觉得属于自己所在的群体
			group_bel2	**Permettono di identificarsi con il proprio gruppo di riferimento**	**They facilitate people's identify with their own reference group**	它们激发了人们对于自己参照群体的认同
			group_bel3	**Costituiscono un comune denominatore con le altre persone del proprio gruppo**	**They are a common denominator with others from people's own group**	它们是群体中人们共同喜欢的
			group_bel4	**Permettono di sviluppare e/o mantenere senso di appartenenza nei confronti del**	**They allow people to develop and/or to maintain a sense of belonging**	它们有益于人们发展/维持对自身群体的归属感

				**proprio gruppo etnico**	**to their own ethnic group**	
